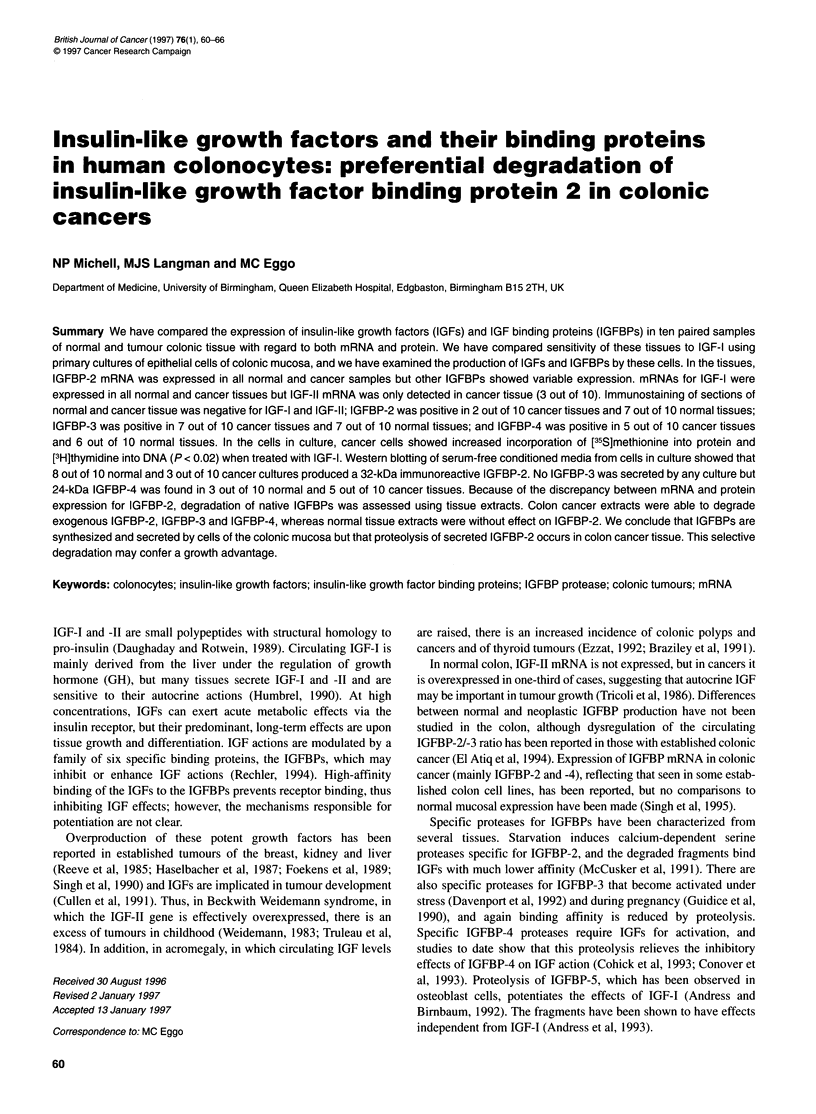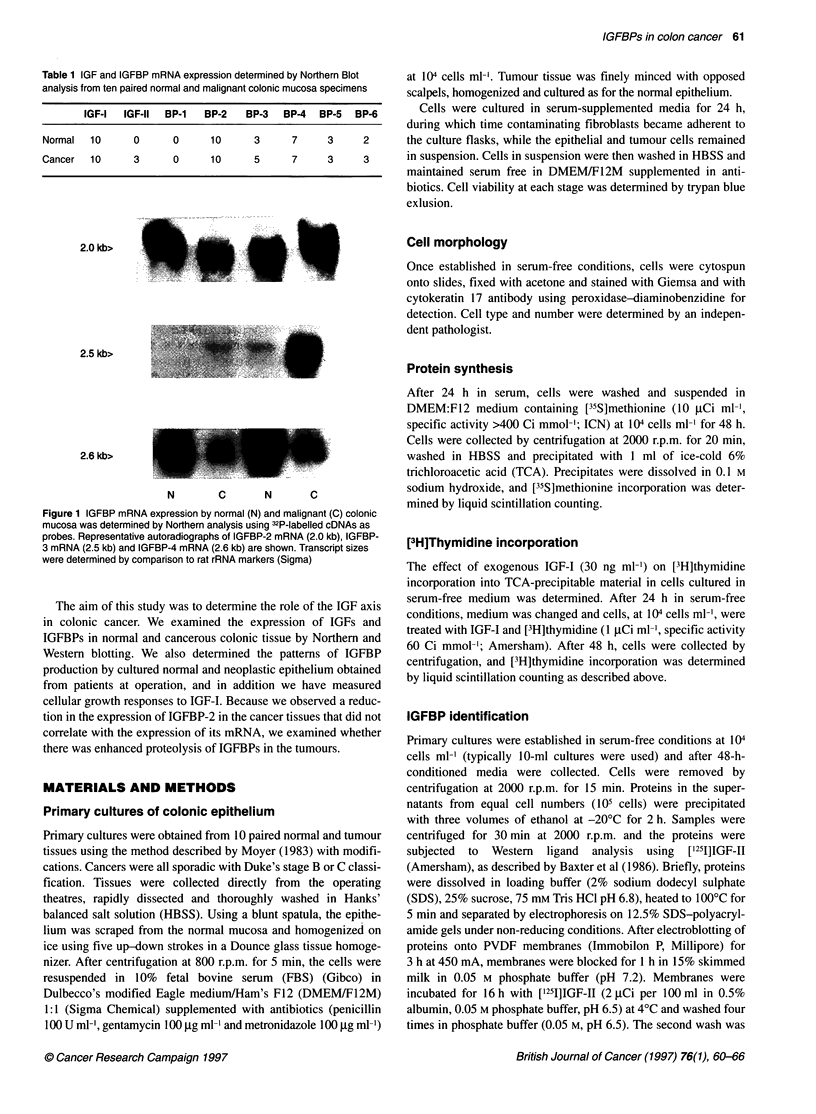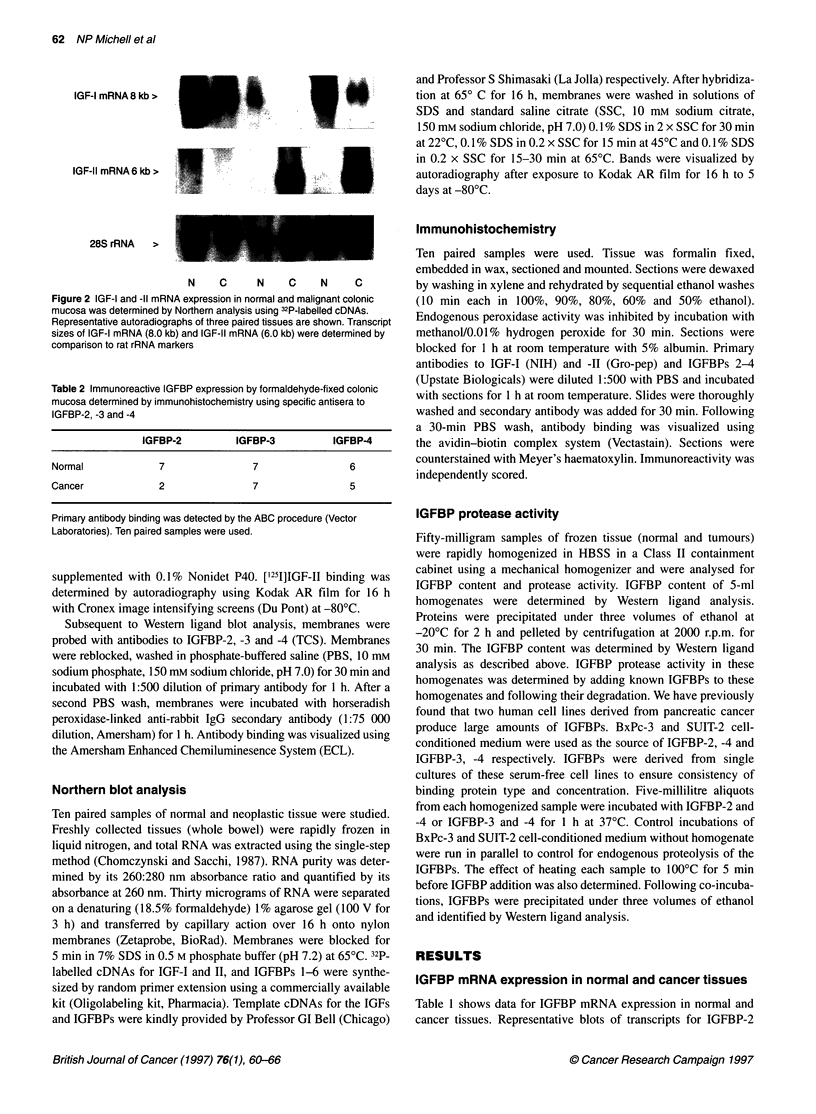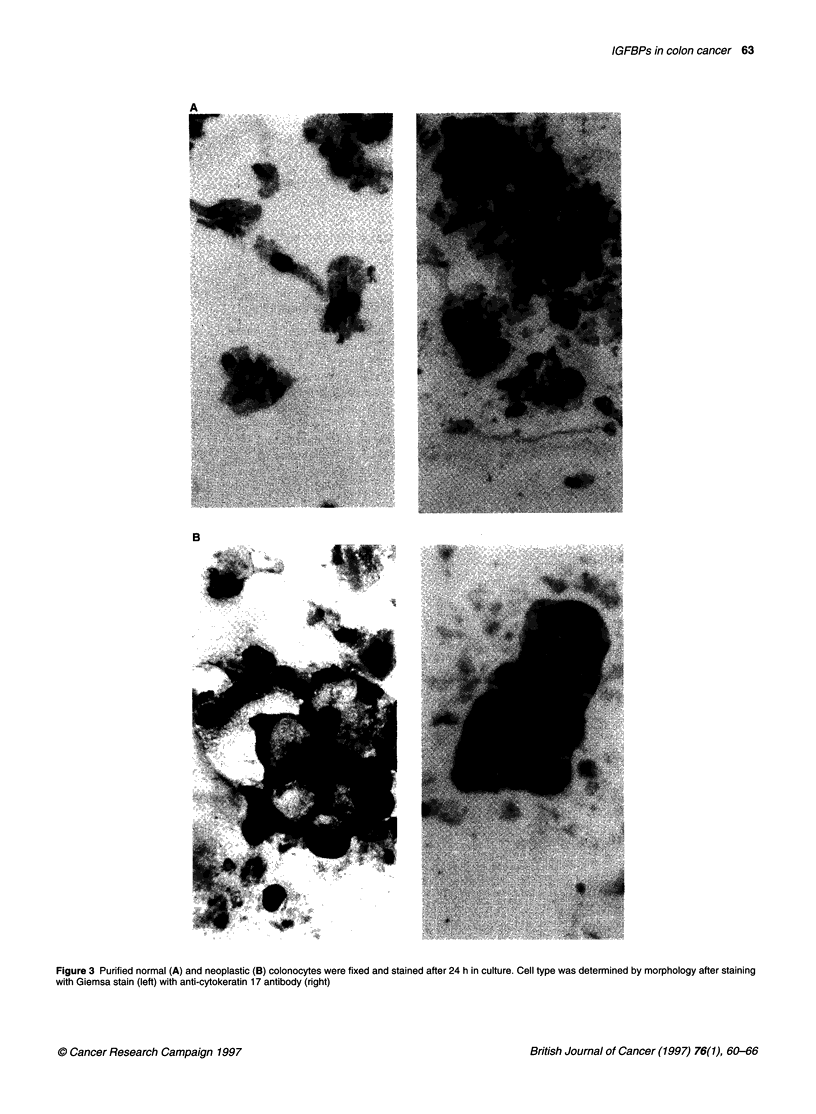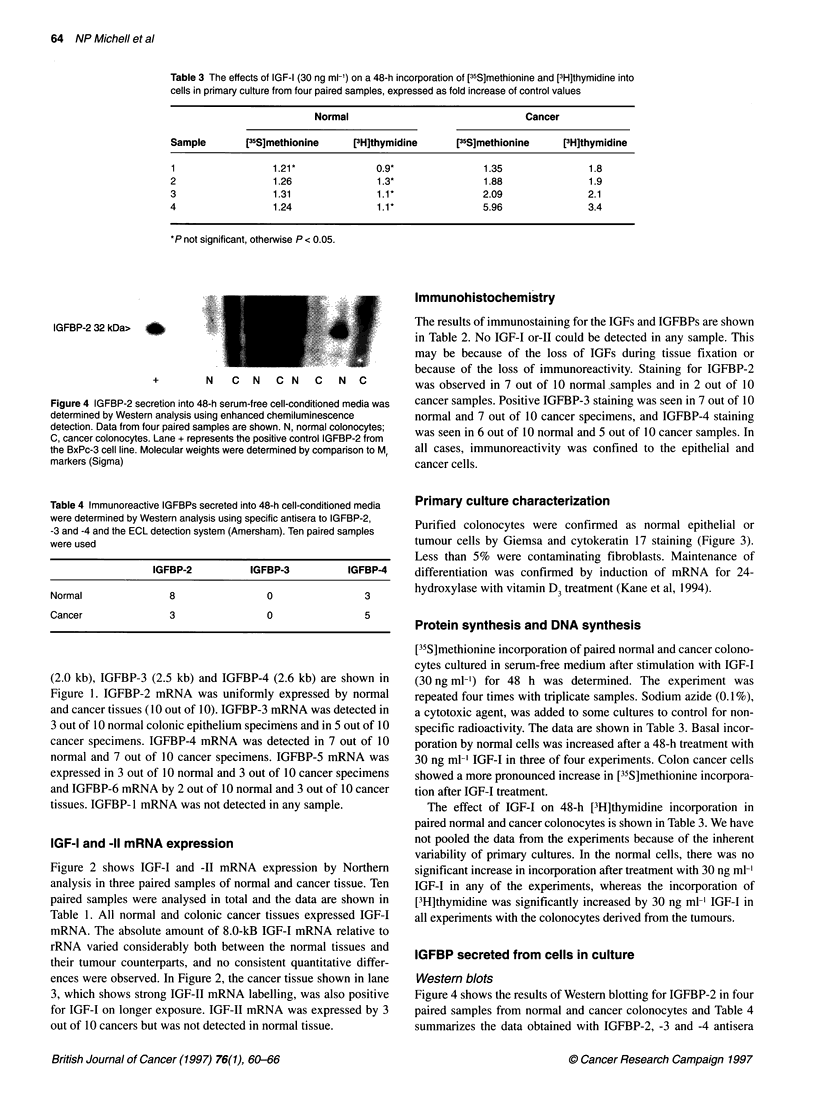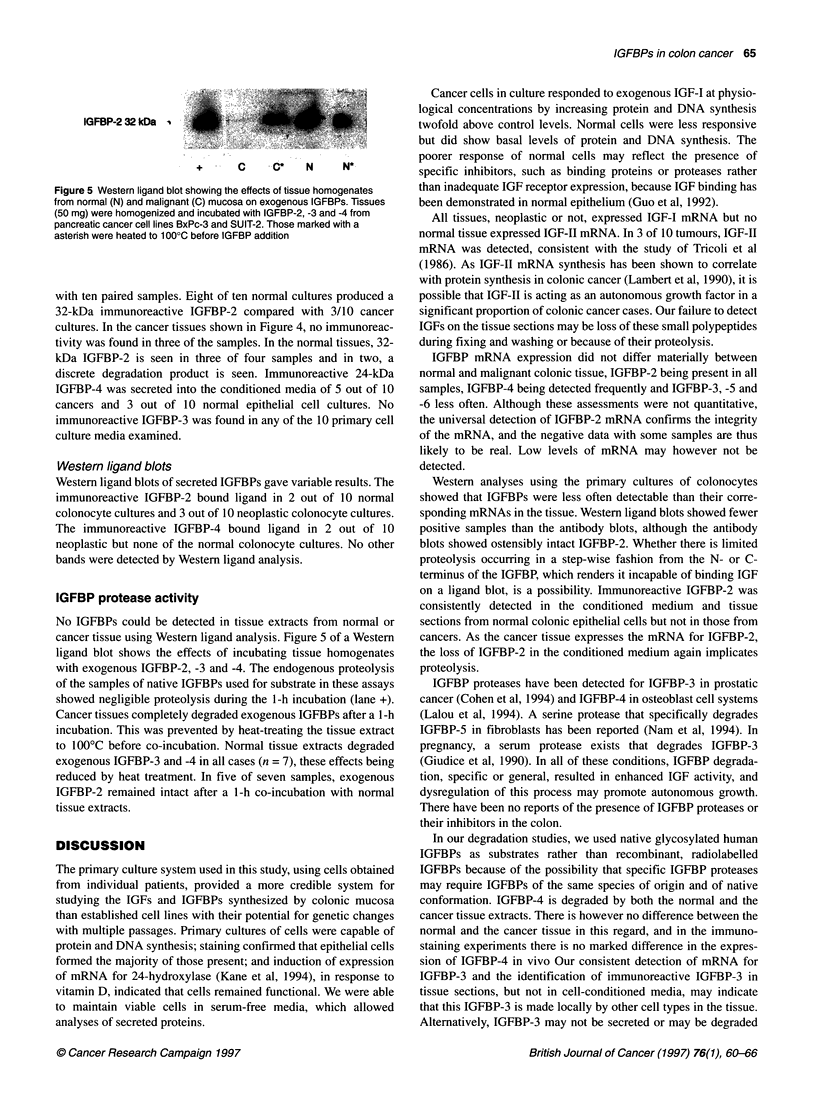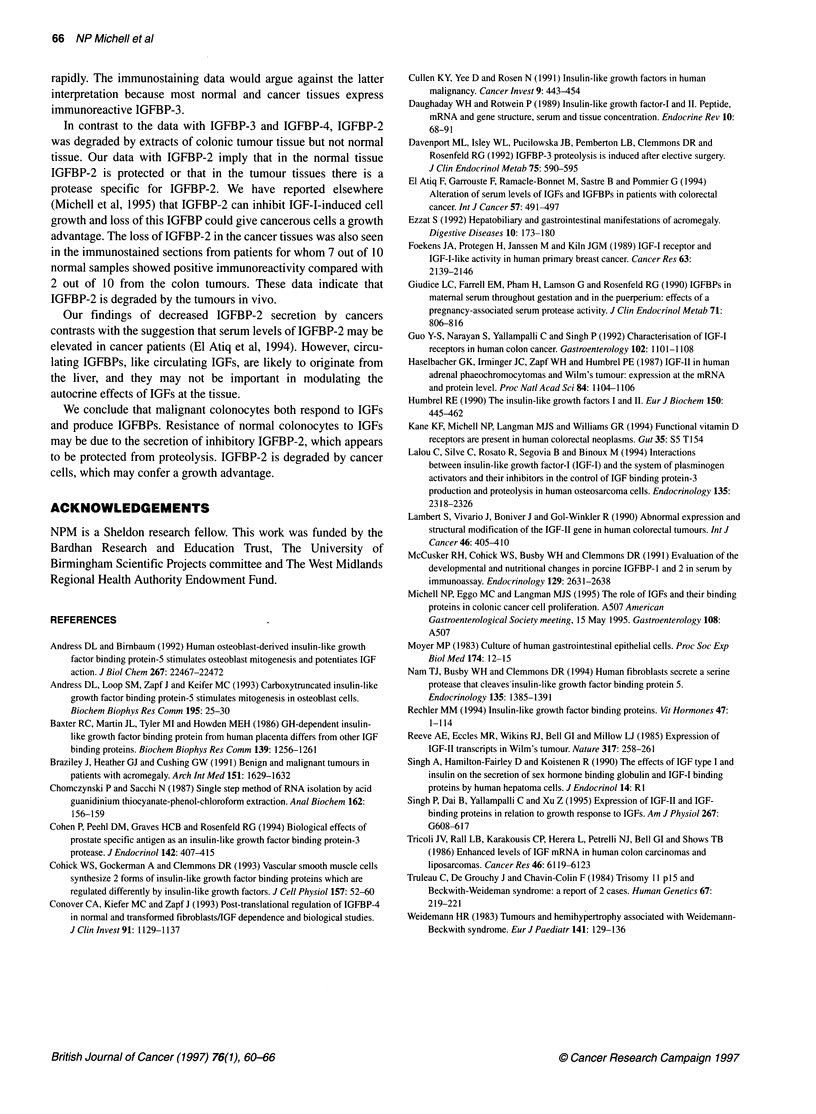# Insulin-like growth factors and their binding proteins in human colonocytes: preferential degradation of insulin-like growth factor binding protein 2 in colonic cancers.

**DOI:** 10.1038/bjc.1997.337

**Published:** 1997

**Authors:** N. P. Michell, M. J. Langman, M. C. Eggo

**Affiliations:** Department of Medicine, University of Birmingham, Queen Elizabeth Hospital, Edgbaston, UK.

## Abstract

**Images:**


British Journal of Cancer (1997) **76**(1), 60–66
© 1997 Cancer Research Campaign

### Insulin-like growth factors and their binding proteins in human colonocytes: preferential degradation of insulin-like growth factor binding protein 2 in colonic cancers

**NP Michell, MJS Langman and MC Eggo**

Department of Medicine, University of Birmingham, Queen Elizabeth Hospital, Edgbaston, Birmingham B15 2TH, UK

**Summary** We have compared the expression of insulin-like growth factors (IGFs) and IGF binding proteins (IGFBPs) in ten paired samples of normal and tumour colonic tissue with regard to both mRNA and protein. We have compared sensitivity of these tissues to IGF-I using primary cultures of epithelial cells of colonic mucosa, and we have examined the production of IGFs and IGFBPs by these cells. In the tissues, IGFBP-2 mRNA was expressed in all normal and cancer samples but other IGFBPs showed variable expression. mRNAs for IGF-I were expressed in all normal and cancer tissues but IGF-II mRNA was only detected in cancer tissue (3 out of 10). Immunostaining of sections of normal and cancer tissue was negative for IGF-I and IGF-II; IGFBP-2 was positive in 2 out of 10 cancer tissues and 7 out of 10 normal tissues; IGFBP-3 was positive in 7 out of 10 cancer tissues and 7 out of 10 normal tissues; and IGFBP-4 was positive in 5 out of 10 cancer tissues and 6 out of 10 normal tissues. In the cells in culture, cancer cells showed increased incorporation of [$^{35}$S]methionine into protein and [$^{3}$H]thymidine into DNA ($P < 0.02$) when treated with IGF-I. Western blotting of serum-free conditioned media from cells in culture showed that 8 out of 10 normal and 3 out of 10 cancer cultures produced a 32-kDa immunoreactive IGFBP-2. No IGFBP-3 was secreted by any culture but 24-kDa IGFBP-4 was found in 3 out of 10 normal and 5 out of 10 cancer tissues. Because of the discrepancy between mRNA and protein expression for IGFBP-2, degradation of native IGFBPs was assessed using tissue extracts. Colon cancer extracts were able to degrade exogenous IGFBP-2, IGFBP-3 and IGFBP-4, whereas normal tissue extracts were without effect on IGFBP-2. We conclude that IGFBPs are synthesized and secreted by cells of the colonic mucosa but that proteolysis of secreted IGFBP-2 occurs in colon cancer tissue. This selective degradation may confer a growth advantage.

**Keywords:** colonocytes; insulin-like growth factors; insulin-like growth factor binding proteins; IGFBP protease; colonic tumours; mRNA

IGF-I and -II are small polypeptides with structural homology to pro-insulin (Daughaday and Rotwein, 1989). Circulating IGF-I is mainly derived from the liver under the regulation of growth hormone (GH), but many tissues secrete IGF-I and -II and are sensitive to their autocrine actions (Humbrel, 1990). At high concentrations, IGFs can exert acute metabolic effects via the insulin receptor, but their predominant, long-term effects are upon tissue growth and differentiation. IGF actions are modulated by a family of six specific binding proteins, the IGFBPs, which may inhibit or enhance IGF actions (Rechler, 1994). High-affinity binding of the IGFs to the IGFBPs prevents receptor binding, thus inhibiting IGF effects; however, the mechanisms responsible for potentiation are not clear.

Overproduction of these potent growth factors has been reported in established tumours of the breast, kidney and liver (Reeve et al, 1985; Haselbacher et al, 1987; Foekens et al, 1989; Singh et al, 1990) and IGFs are implicated in tumour development (Cullen et al, 1991). Thus, in Beckwith Weidemann syndrome, in which the IGF-II gene is effectively overexpressed, there is an excess of tumours in childhood (Weidemann, 1983; Truleau et al, 1984). In addition, in acromegaly, in which circulating IGF levels are raised, there is an increased incidence of colonic polyps and cancers and of thyroid tumours (Ezzat, 1992; Braziley et al, 1991).

*Received 30 August 1996*
*Revised 2 January 1997*
*Accepted 13 January 1997*

*Correspondence to:* MC Eggo

In normal colon, IGF-II mRNA is not expressed, but in cancers it is overexpressed in one-third of cases, suggesting that autocrine IGF may be important in tumour growth (Tricoli et al, 1986). Differences between normal and neoplastic IGFBP production have not been studied in the colon, although dysregulation of the circulating IGFBP-2/-3 ratio has been reported in those with established colonic cancer (El Atiq et al, 1994). Expression of IGFBP mRNA in colonic cancer (mainly IGFBP-2 and -4), reflecting that seen in some established colon cell lines, has been reported, but no comparisons to normal mucosal expression have been made (Singh et al, 1995).

Specific proteases for IGFBPs have been characterized from several tissues. Starvation induces calcium-dependent serine proteases specific for IGFBP-2, and the degraded fragments bind IGFs with much lower affinity (McCusker et al, 1991). There are also specific proteases for IGFBP-3 that become activated under stress (Davenport et al, 1992) and during pregnancy (Guidice et al, 1990), and again binding affinity is reduced by proteolysis. Specific IGFBP-4 proteases require IGFs for activation, and studies to date show that this proteolysis relieves the inhibitory effects of IGFBP-4 on IGF action (Cohick et al, 1993; Conover et al, 1993). Proteolysis of IGFBP-5, which has been observed in osteoblast cells, potentiates the effects of IGF-I (Andress and Birnbaum, 1992). The fragments have been shown to have effects independent from IGF-I (Andress et al, 1993).

**Table 1** IGF and IGFBP mRNA expression determined by Northern Blot analysis from ten paired normal and malignant colonic mucosa specimens

| | IGF-I | IGF-II | BP-1 | BP-2 | BP-3 | BP-4 | BP-5 | BP-6 |
|---|---|---|---|---|---|---|---|---|
| Normal | 10 | 0 | 0 | 10 | 3 | 7 | 3 | 2 |
| Cancer | 10 | 3 | 0 | 10 | 5 | 7 | 3 | 3 |

**Figure 1** IGFBP mRNA expression by normal (N) and malignant (C) colonic mucosa was determined by Northern analysis using $^{32}$P-labelled cDNAs as probes. Representative autoradiographs of IGFBP-2 mRNA (2.0 kb), IGFBP-3 mRNA (2.5 kb) and IGFBP-4 mRNA (2.6 kb) are shown. Transcript sizes were determined by comparison to rat rRNA markers (Sigma)

The aim of this study was to determine the role of the IGF axis in colonic cancer. We examined the expression of IGFs and IGFBPs in normal and cancerous colonic tissue by Northern and Western blotting. We also determined the patterns of IGFBP production by cultured normal and neoplastic epithelium obtained from patients at operation, and in addition we have measured cellular growth responses to IGF-I. Because we observed a reduction in the expression of IGFBP-2 in the cancer tissues that did not correlate with the expression of its mRNA, we examined whether there was enhanced proteolysis of IGFBPs in the tumours.

### MATERIALS AND METHODS

### Primary cultures of colonic epithelium

Primary cultures were obtained from 10 paired normal and tumour tissues using the method described by Moyer (1983) with modifications. Cancers were all sporadic with Duke's stage B or C classification. Tissues were collected directly from the operating theatres, rapidly dissected and thoroughly washed in Hanks' balanced salt solution (HBSS). Using a blunt spatula, the epithelium was scraped from the normal mucosa and homogenized on ice using five up–down strokes in a Dounce glass tissue homogenizer. After centrifugation at 800 r.p.m. for 5 min, the cells were resuspended in 10% fetal bovine serum (FBS) (Gibco) in Dulbecco's modified Eagle medium/Ham's F12 (DMEM/F12M) 1:1 (Sigma Chemical) supplemented with antibiotics (penicillin 100 U ml$^{-1}$, gentamycin 100 µg ml$^{-1}$ and metronidazole 100 µg ml$^{-1}$) at $10^4$ cells ml$^{-1}$. Tumour tissue was finely minced with opposed scalpels, homogenized and cultured as for the normal epithelium.

Cells were cultured in serum-supplemented media for 24 h, during which time contaminating fibroblasts became adherent to the culture flasks, while the epithelial and tumour cells remained in suspension. Cells in suspension were then washed in HBSS and maintained serum free in DMEM/F12M supplemented in antibiotics. Cell viability at each stage was determined by trypan blue exlusion.

### Cell morphology

Once established in serum-free conditions, cells were cytospun onto slides, fixed with acetone and stained with Giemsa and with cytokeratin 17 antibody using peroxidase–diaminobenzidine for detection. Cell type and number were determined by an independent pathologist.

### Protein synthesis

After 24 h in serum, cells were washed and suspended in DMEM:F12 medium containing [$^{35}$S]methionine (10 µCi ml$^{-1}$, specific activity >400 Ci mmol$^{-1}$; ICN) at $10^4$ cells ml$^{-1}$ for 48 h. Cells were collected by centrifugation at 2000 r.p.m. for 20 min, washed in HBSS and precipitated with 1 ml of ice-cold 6% trichloroacetic acid (TCA). Precipitates were dissolved in 0.1 M sodium hydroxide, and [$^{35}$S]methionine incorporation was determined by liquid scintillation counting.

### [$^3$H]Thymidine incorporation

The effect of exogenous IGF-I (30 ng ml$^{-1}$) on [$^3$H]thymidine incorporation into TCA-precipitable material in cells cultured in serum-free medium was determined. After 24 h in serum-free conditions, medium was changed and cells, at $10^4$ cells ml$^{-1}$, were treated with IGF-I and [$^3$H]thymidine (1 µCi ml$^{-1}$, specific activity 60 Ci mmol$^{-1}$; Amersham). After 48 h, cells were collected by centrifugation, and [$^3$H]thymidine incorporation was determined by liquid scintillation counting as described above.

### IGFBP identification

Primary cultures were established in serum-free conditions at $10^4$ cells ml$^{-1}$ (typically 10-ml cultures were used) and after 48-h-conditioned media were collected. Cells were removed by centrifugation at 2000 r.p.m. for 15 min. Proteins in the supernatants from equal cell numbers ($10^5$ cells) were precipitated with three volumes of ethanol at –20°C for 2 h. Samples were centrifuged for 30 min at 2000 r.p.m. and the proteins were subjected to Western ligand analysis using [$^{125}$I]IGF-II (Amersham), as described by Baxter et al (1986). Briefly, proteins were dissolved in loading buffer (2% sodium dodecyl sulphate (SDS), 25% sucrose, 75 mM Tris HCl pH 6.8), heated to 100°C for 5 min and separated by electrophoresis on 12.5% SDS–polyacrylamide gels under non-reducing conditions. After electroblotting of proteins onto PVDF membranes (Immobilon P, Millipore) for 3 h at 450 mA, membranes were blocked for 1 h in 15% skimmed milk in 0.05 M phosphate buffer (pH 7.2). Membranes were incubated for 16 h with [$^{125}$I]IGF-II (2 µCi per 100 ml in 0.5% albumin, 0.05 M phosphate buffer, pH 6.5) at 4°C and washed four times in phosphate buffer (0.05 M, pH 6.5). The second wash was

© Cancer Research Campaign 1997

**Figure 2** IGF-I and -II mRNA expression in normal and malignant colonic mucosa was determined by Northern analysis using $^{32}P$-labelled cDNAs. Representative autoradiographs of three paired tissues are shown. Transcript sizes of IGF-I mRNA (8.0 kb) and IGF-II mRNA (6.0 kb) were determined by comparison to rat rRNA markers

**Table 2** Immunoreactive IGFBP expression by formaldehyde-fixed colonic mucosa determined by immunohistochemistry using specific antisera to IGFBP-2, -3 and -4

| | IGFBP-2 | IGFBP-3 | IGFBP-4 |
|---|---|---|---|
| Normal | 7 | 7 | 6 |
| Cancer | 2 | 7 | 5 |

Primary antibody binding was detected by the ABC procedure (Vector Laboratories). Ten paired samples were used.

supplemented with 0.1% Nonidet P40. [$^{125}$I]IGF-II binding was determined by autoradiography using Kodak AR film for 16 h with Cronex image intensifying screens (Du Pont) at –80°C.

Subsequent to Western ligand blot analysis, membranes were probed with antibodies to IGFBP-2, -3 and -4 (TCS). Membranes were reblocked, washed in phosphate-buffered saline (PBS, 10 mM sodium phosphate, 150 mM sodium chloride, pH 7.0) for 30 min and incubated with 1:500 dilution of primary antibody for 1 h. After a second PBS wash, membranes were incubated with horseradish peroxidase-linked anti-rabbit IgG secondary antibody (1:75 000 dilution, Amersham) for 1 h. Antibody binding was visualized using the Amersham Enhanced Chemiluminesence System (ECL).

### Northern blot analysis

Ten paired samples of normal and neoplastic tissue were studied. Freshly collected tissues (whole bowel) were rapidly frozen in liquid nitrogen, and total RNA was extracted using the single-step method (Chomczynski and Sacchi, 1987). RNA purity was determined by its 260:280 nm absorbance ratio and quantified by its absorbance at 260 nm. Thirty micrograms of RNA were separated on a denaturing (18.5% formaldehyde) 1% agarose gel (100 V for 3 h) and transferred by capillary action over 16 h onto nylon membranes (Zetaprobe, BioRad). Membranes were blocked for 5 min in 7% SDS in 0.5 M phosphate buffer (pH 7.2) at 65°C. $^{32}P$-labelled cDNAs for IGF-I and II, and IGFBPs 1–6 were synthesized by random primer extension using a commercially available kit (Oligolabeling kit, Pharmacia). Template cDNAs for the IGFs and IGFBPs were kindly provided by Professor GI Bell (Chicago) and Professor S Shimasaki (La Jolla) respectively. After hybridization at 65° C for 16 h, membranes were washed in solutions of SDS and standard saline citrate (SSC, 10 mM sodium citrate, 150 mM sodium chloride, pH 7.0) 0.1% SDS in 2 × SSC for 30 min at 22°C, 0.1% SDS in 0.2 × SSC for 15 min at 45°C and 0.1% SDS in 0.2 × SSC for 15–30 min at 65°C. Bands were visualized by autoradiography after exposure to Kodak AR film for 16 h to 5 days at –80°C.

### Immunohistochemistry

Ten paired samples were used. Tissue was formalin fixed, embedded in wax, sectioned and mounted. Sections were dewaxed by washing in xylene and rehydrated by sequential ethanol washes (10 min each in 100%, 90%, 80%, 60% and 50% ethanol). Endogenous peroxidase activity was inhibited by incubation with methanol/0.01% hydrogen peroxide for 30 min. Sections were blocked for 1 h at room temperature with 5% albumin. Primary antibodies to IGF-I (NIH) and -II (Gro-pep) and IGFBPs 2–4 (Upstate Biologicals) were diluted 1:500 with PBS and incubated with sections for 1 h at room temperature. Slides were thoroughly washed and secondary antibody was added for 30 min. Following a 30-min PBS wash, antibody binding was visualized using the avidin–biotin complex system (Vectastain). Sections were counterstained with Meyer's haematoxylin. Immunoreactivity was independently scored.

### IGFBP protease activity

Fifty-milligram samples of frozen tissue (normal and tumours) were rapidly homogenized in HBSS in a Class II containment cabinet using a mechanical homogenizer and were analysed for IGFBP content and protease activity. IGFBP content of 5-ml homogenates were determined by Western ligand analysis. Proteins were precipitated under three volumes of ethanol at –20°C for 2 h and pelleted by centrifugation at 2000 r.p.m. for 30 min. The IGFBP content was determined by Western ligand analysis as described above. IGFBP protease activity in these homogenates was determined by adding known IGFBPs to these homogenates and following their degradation. We have previously found that two human cell lines derived from pancreatic cancer produce large amounts of IGFBPs. BxPc-3 and SUIT-2 cell-conditioned medium were used as the source of IGFBP-2, -4 and IGFBP-3, -4 respectively. IGFBPs were derived from single cultures of these serum-free cell lines to ensure consistency of binding protein type and concentration. Five-millilitre aliquots from each homogenized sample were incubated with IGFBP-2 and -4 or IGFBP-3 and -4 for 1 h at 37°C. Control incubations of BxPc-3 and SUIT-2 cell-conditioned medium without homogenate were run in parallel to control for endogenous proteolysis of the IGFBPs. The effect of heating each sample to 100°C for 5 min before IGFBP addition was also determined. Following co-incubations, IGFBPs were precipitated under three volumes of ethanol and identified by Western ligand analysis.

### RESULTS

### IGFBP mRNA expression in normal and cancer tissues

Table 1 shows data for IGFBP mRNA expression in normal and cancer tissues. Representative blots of transcripts for IGFBP-2

 © Cancer Research Campaign 1997

A

B

Figure 3 Purified normal (A) and neoplastic (B) colonocytes were fixed and stained after 24 h in culture. Cell type was determined by morphology after staining with Giemsa stain (left) with anti-cytokeratin 17 antibody (right)

© Cancer Research Campaign 1997 

**Table 3** The effects of IGF-I (30 ng ml$^{-1}$) on a 48-h incorporation of [$^{35}$S]methionine and [$^{3}$H]thymidine into cells in primary culture from four paired samples, expressed as fold increase of control values

| | Normal | | Cancer | |
|---|---|---|---|---|
| Sample | [$^{35}$S]methionine | [$^{3}$H]thymidine | [$^{35}$S]methionine | [$^{3}$H]thymidine |
| 1 | 1.21* | 0.9* | 1.35 | 1.8 |
| 2 | 1.26 | 1.3* | 1.88 | 1.9 |
| 3 | 1.31 | 1.1* | 2.09 | 2.1 |
| 4 | 1.24 | 1.1* | 5.96 | 3.4 |

*$P$ not significant, otherwise $P < 0.05$.

**Figure 4** IGFBP-2 secretion into 48-h serum-free cell-conditioned media was determined by Western analysis using enhanced chemiluminescence detection. Data from four paired samples are shown. N, normal colonocytes; C, cancer colonocytes. Lane + represents the positive control IGFBP-2 from the BxPc-3 cell line. Molecular weights were determined by comparison to $M_r$ markers (Sigma)

**Table 4** Immunoreactive IGFBPs secreted into 48-h cell-conditioned media were determined by Western analysis using specific antisera to IGFBP-2, -3 and -4 and the ECL detection system (Amersham). Ten paired samples were used

| | IGFBP-2 | IGFBP-3 | IGFBP-4 |
|---|---|---|---|
| Normal | 8 | 0 | 3 |
| Cancer | 3 | 0 | 5 |

(2.0 kb), IGFBP-3 (2.5 kb) and IGFBP-4 (2.6 kb) are shown in Figure 1. IGFBP-2 mRNA was uniformly expressed by normal and cancer tissues (10 out of 10). IGFBP-3 mRNA was detected in 3 out of 10 normal colonic epithelium specimens and in 5 out of 10 cancer specimens. IGFBP-4 mRNA was detected in 7 out of 10 normal and 7 out of 10 cancer specimens. IGFBP-5 mRNA was expressed in 3 out of 10 normal and 3 out of 10 cancer specimens and IGFBP-6 mRNA by 2 out of 10 normal and 3 out of 10 cancer tissues. IGFBP-1 mRNA was not detected in any sample.

### IGF-I and -II mRNA expression

Figure 2 shows IGF-I and -II mRNA expression by Northern analysis in three paired samples of normal and cancer tissue. Ten paired samples were analysed in total and the data are shown in Table 1. All normal and colonic cancer tissues expressed IGF-I mRNA. The absolute amount of 8.0-kB IGF-I mRNA relative to rRNA varied considerably both between the normal tissues and their tumour counterparts, and no consistent quantitative differences were observed. In Figure 2, the cancer tissue shown in lane 3, which shows strong IGF-II mRNA labelling, was also positive for IGF-I on longer exposure. IGF-II mRNA was expressed by 3 out of 10 cancers but was not detected in normal tissue.

### Immunohistochemistry

The results of immunostaining for the IGFs and IGFBPs are shown in Table 2. No IGF-I or-II could be detected in any sample. This may be because of the loss of IGFs during tissue fixation or because of the loss of immunoreactivity. Staining for IGFBP-2 was observed in 7 out of 10 normal samples and in 2 out of 10 cancer samples. Positive IGFBP-3 staining was seen in 7 out of 10 normal and 7 out of 10 cancer specimens, and IGFBP-4 staining was seen in 6 out of 10 normal and 5 out of 10 cancer samples. In all cases, immunoreactivity was confined to the epithelial and cancer cells.

### Primary culture characterization

Purified colonocytes were confirmed as normal epithelial or tumour cells by Giemsa and cytokeratin 17 staining (Figure 3). Less than 5% were contaminating fibroblasts. Maintenance of differentiation was confirmed by induction of mRNA for 24-hydroxylase with vitamin $D_3$ treatment (Kane et al, 1994).

### Protein synthesis and DNA synthesis

[$^{35}$S]methionine incorporation of paired normal and cancer colonocytes cultured in serum-free medium after stimulation with IGF-I (30 ng ml$^{-1}$) for 48 h was determined. The experiment was repeated four times with triplicate samples. Sodium azide (0.1%), a cytotoxic agent, was added to some cultures to control for non-specific radioactivity. The data are shown in Table 3. Basal incorporation by normal cells was increased after a 48-h treatment with 30 ng ml$^{-1}$ IGF-I in three of four experiments. Colon cancer cells showed a more pronounced increase in [$^{35}$S]methionine incorporation after IGF-I treatment.

The effect of IGF-I on 48-h [$^{3}$H]thymidine incorporation in paired normal and cancer colonocytes is shown in Table 3. We have not pooled the data from the experiments because of the inherent variability of primary cultures. In the normal cells, there was no significant increase in incorporation after treatment with 30 ng ml$^{-1}$ IGF-I in any of the experiments, whereas the incorporation of [$^{3}$H]thymidine was significantly increased by 30 ng ml$^{-1}$ IGF-I in all experiments with the colonocytes derived from the tumours.

### IGFBP secreted from cells in culture

### *Western blots*

Figure 4 shows the results of Western blotting for IGFBP-2 in four paired samples from normal and cancer colonocytes and Table 4 summarizes the data obtained with IGFBP-2, -3 and -4 antisera

 © Cancer Research Campaign 1997

**Figure 5** Western ligand blot showing the effects of tissue homogenates from normal (N) and malignant (C) mucosa on exogenous IGFBPs. Tissues (50 mg) were homogenized and incubated with IGFBP-2, -3 and -4 from pancreatic cancer cell lines BxPc-3 and SUIT-2. Those marked with a asterish were heated to 100°C before IGFBP addition with ten paired samples. Eight of ten normal cultures produced a 32-kDa immunoreactive IGFBP-2 compared with 3/10 cancer cultures. In the cancer tissues shown in Figure 4, no immunoreactivity was found in three of the samples. In the normal tissues, 32-kDa IGFBP-2 is seen in three of four samples and in two, a discrete degradation product is seen. Immunoreactive 24-kDa IGFBP-4 was secreted into the conditioned media of 5 out of 10 cancers and 3 out of 10 normal epithelial cell cultures. No immunoreactive IGFBP-3 was found in any of the 10 primary cell culture media examined.

### *Western ligand blots*

Western ligand blots of secreted IGFBPs gave variable results. The immunoreactive IGFBP-2 bound ligand in 2 out of 10 normal colonocyte cultures and 3 out of 10 neoplastic colonocyte cultures. The immunoreactive IGFBP-4 bound ligand in 2 out of 10 neoplastic but none of the normal colonocyte cultures. No other bands were detected by Western ligand analysis.

### IGFBP protease activity

No IGFBPs could be detected in tissue extracts from normal or cancer tissue using Western ligand analysis. Figure 5 of a Western ligand blot shows the effects of incubating tissue homogenates with exogenous IGFBP-2, -3 and -4. The endogenous proteolysis of the samples of native IGFBPs used for substrate in these assays showed negligible proteolysis during the 1-h incubation (lane +). Cancer tissues completely degraded exogenous IGFBPs after a 1-h incubation. This was prevented by heat-treating the tissue extract to 100°C before co-incubation. Normal tissue extracts degraded exogenous IGFBP-3 and -4 in all cases ($n$ = 7), these effects being reduced by heat treatment. In five of seven samples, exogenous IGFBP-2 remained intact after a 1-h co-incubation with normal tissue extracts.

### DISCUSSION

The primary culture system used in this study, using cells obtained from individual patients, provided a more credible system for studying the IGFs and IGFBPs synthesized by colonic mucosa than established cell lines with their potential for genetic changes with multiple passages. Primary cultures of cells were capable of protein and DNA synthesis; staining confirmed that epithelial cells formed the majority of those present; and induction of expression of mRNA for 24-hydroxylase (Kane et al, 1994), in response to vitamin D, indicated that cells remained functional. We were able to maintain viable cells in serum-free media, which allowed analyses of secreted proteins.

Cancer cells in culture responded to exogenous IGF-I at physiological concentrations by increasing protein and DNA synthesis twofold above control levels. Normal cells were less responsive but did show basal levels of protein and DNA synthesis. The poorer response of normal cells may reflect the presence of specific inhibitors, such as binding proteins or proteases rather than inadequate IGF receptor expression, because IGF binding has been demonstrated in normal epithelium (Guo et al, 1992).

All tissues, neoplastic or not, expressed IGF-I mRNA but no normal tissue expressed IGF-II mRNA. In 3 of 10 tumours, IGF-II mRNA was detected, consistent with the study of Tricoli et al (1986). As IGF-II mRNA synthesis has been shown to correlate with protein synthesis in colonic cancer (Lambert et al, 1990), it is possible that IGF-II is acting as an autonomous growth factor in a significant proportion of colonic cancer cases. Our failure to detect IGFs on the tissue sections may be loss of these small polypeptides during fixing and washing or because of their proteolysis.

IGFBP mRNA expression did not differ materially between normal and malignant colonic tissue, IGFBP-2 being present in all samples, IGFBP-4 being detected frequently and IGFBP-3, -5 and -6 less often. Although these assessments were not quantitative, the universal detection of IGFBP-2 mRNA confirms the integrity of the mRNA, and the negative data with some samples are thus likely to be real. Low levels of mRNA may however not be detected.

Western analyses using the primary cultures of colonocytes showed that IGFBPs were less often detectable than their corresponding mRNAs in the tissue. Western ligand blots showed fewer positive samples than the antibody blots, although the antibody blots showed ostensibly intact IGFBP-2. Whether there is limited proteolysis occurring in a step-wise fashion from the N- or C-terminus of the IGFBP, which renders it incapable of binding IGF on a ligand blot, is a possibility. Immunoreactive IGFBP-2 was consistently detected in the conditioned medium and tissue sections from normal colonic epithelial cells but not in those from cancers. As the cancer tissue expresses the mRNA for IGFBP-2, the loss of IGFBP-2 in the conditioned medium again implicates proteolysis.

IGFBP proteases have been detected for IGFBP-3 in prostatic cancer (Cohen et al, 1994) and IGFBP-4 in osteoblast cell systems (Lalou et al, 1994). A serine protease that specifically degrades IGFBP-5 in fibroblasts has been reported (Nam et al, 1994). In pregnancy, a serum protease exists that degrades IGFBP-3 (Giudice et al, 1990). In all of these conditions, IGFBP degradation, specific or general, resulted in enhanced IGF activity, and dysregulation of this process may promote autonomous growth. There have been no reports of the presence of IGFBP proteases or their inhibitors in the colon.

In our degradation studies, we used native glycosylated human IGFBPs as substrates rather than recombinant, radiolabelled IGFBPs because of the possibility that specific IGFBP proteases may require IGFBPs of the same species of origin and of native conformation. IGFBP-4 is degraded by both the normal and the cancer tissue extracts. There is however no difference between the normal and the cancer tissue in this regard, and in the immunostaining experiments there is no marked difference in the expression of IGFBP-4 in vivo Our consistent detection of mRNA for IGFBP-3 and the identification of immunoreactive IGFBP-3 in tissue sections, but not in cell-conditioned media, may indicate that this IGFBP-3 is made locally by other cell types in the tissue. Alternatively, IGFBP-3 may not be secreted or may be degraded

© Cancer Research Campaign 1997

rapidly. The immunostaining data would argue against the latter interpretation because most normal and cancer tissues express immunoreactive IGFBP-3.

In contrast to the data with IGFBP-3 and IGFBP-4, IGFBP-2 was degraded by extracts of colonic tumour tissue but not normal tissue. Our data with IGFBP-2 imply that in the normal tissue IGFBP-2 is protected or that in the tumour tissues there is a protease specific for IGFBP-2. We have reported elsewhere (Michell et al, 1995) that IGFBP-2 can inhibit IGF-I-induced cell growth and loss of this IGFBP could give cancerous cells a growth advantage. The loss of IGFBP-2 in the cancer tissues was also seen in the immunostained sections from patients for whom 7 out of 10 normal samples showed positive immunoreactivity compared with 2 out of 10 from the colon tumours. These data indicate that IGFBP-2 is degraded by the tumours in vivo.

Our findings of decreased IGFBP-2 secretion by cancers contrasts with the suggestion that serum levels of IGFBP-2 may be elevated in cancer patients (El Atiq et al, 1994). However, circulating IGFBPs, like circulating IGFs, are likely to originate from the liver, and they may not be important in modulating the autocrine effects of IGFs at the tissue.

We conclude that malignant colonocytes both respond to IGFs and produce IGFBPs. Resistance of normal colonocytes to IGFs may be due to the secretion of inhibitory IGFBP-2, which appears to be protected from proteolysis. IGFBP-2 is degraded by cancer cells, which may confer a growth advantage.

### ACKNOWLEDGEMENTS

NPM is a Sheldon research fellow. This work was funded by the Bardhan Research and Education Trust, The University of Birmingham Scientific Projects committee and The West Midlands Regional Health Authority Endowment Fund.

### REFERENCES

Andress DL and Birnbaum (1992) Human osteoblast-derived insulin-like growth factor binding protein-5 stimulates osteoblast mitogenesis and potentiates IGF action. *J Biol Chem* **267**: 22467–22472

Andress DL, Loop SM, Zapf J and Keifer MC (1993) Carboxytruncated insulin-like growth factor binding protein-5 stimulates mitogenesis in osteoblast cells. *Biochem Biophys Res Comm* **195**: 25–30

Baxter RC, Martin JL, Tyler MI and Howden MEH (1986) GH-dependent insulin-like growth factor binding protein from human placenta differs from other IGF binding proteins. *Biochem Biophys Res Comm* **139**: 1256–1261

Braziley J, Heather GJ and Cushing GW (1991) Benign and malignant tumours in patients with acromegaly. *Arch Int Med* **151**: 1629–1632

Chomczynski P and Sacchi N (1987) Single step method of RNA isolation by acid guanidinium thiocyanate-phenol-chloroform extraction. *Anal Biochem* **162**: 156–159

Cohen P, Peehl DM, Graves HCB and Rosenfeld RG (1994) Biological effects of prostate specific antigen as an insulin-like growth factor binding protein-3 protease. *J Endocrinol* **142**: 407–415

Cohick WS, Gockerman A and Clemmons DR (1993) Vascular smooth muscle cells synthesize 2 forms of insulin-like growth factor binding proteins which are regulated differently by insulin-like growth factors. *J Cell Physiol* **157**: 52–60

Conover CA, Kiefer MC and Zapf J (1993) Post-translational regulation of IGFBP-4 in normal and transformed fibroblasts/IGF dependence and biological studies. *J Clin Invest* **91**: 1129–1137

Cullen KY, Yee D and Rosen N (1991) Insulin-like growth factors in human malignancy. *Cancer Invest* **9**: 443–454

Daughaday WH and Rotwein P (1989) Insulin-like growth factor-I and II. Peptide, mRNA and gene structure, serum and tissue concentration. *Endocrine Rev* **10**: 68–91

Davenport ML, Isley WL, Pucilowska JB, Pemberton LB, Clemmons DR and Rosenfeld RG (1992) IGFBP-3 proteolysis is induced after elective surgery. *J Clin Endocrinol Metab* **75**: 590–595

El Atiq F, Garrouste F, Ramacle-Bonnet M, Sastre B and Pommier G (1994) Alteration of serum levels of IGFs and IGFBPs in patients with colorectal cancer. *Int J Cancer* **57**: 491–497

Ezzat S (1992) Hepatobiliary and gastrointestinal manifestations of acromegaly. *Digestive Diseases* **10**: 173–180

Foekens JA, Protegen H, Janssen M and Kiln JGM (1989) IGF-I receptor and IGF-I-like activity in human primary breast cancer. *Cancer Res* **63**: 2139–2146

Giudice LC, Farrell EM, Pham H, Lamson G and Rosenfeld RG (1990) IGFBPs in maternal serum throughout gestation and in the puerperium: effects of a pregnancy-associated serum protease activity. *J Clin Endocrinol Metab* **71**: 806–816

Guo Y-S, Narayan S, Yallampalli C and Singh P (1992) Characterisation of IGF-I receptors in human colon cancer. *Gastroenterology* **102**: 1101–1108

Haselbacher GK, Irminger JC, Zapf WH and Humbrel PE (1987) IGF-II in human adrenal phaeochromocytomas and Wilm's tumour: expression at the mRNA and protein level. *Proc Natl Acad Sci* **84**: 1104–1106

Humbrel RE (1990) The insulin-like growth factors I and II. *Eur J Biochem* **150**: 445–462

Kane KF, Michell NP, Langman MJS and Williams GR (1994) Functional vitamin D receptors are present in human colorectal neoplasms. *Gut* **35**: S5 T154

Lalou C, Silve C, Rosato R, Segovia B and Binoux M (1994) Interactions between insulin-like growth factor-I (IGF-I) and the system of plasminogen activators and their inhibitors in the control of IGF binding protein-3 production and proteolysis in human osteosarcoma cells. *Endocrinology* **135**: 2318–2326

Lambert S, Vivario J, Boniver J and Gol-Winkler R (1990) Abnormal expression and structural modification of the IGF-II gene in human colorectal tumours. *Int J Cancer* **46**: 405–410

McCusker RH, Cohick WS, Busby WH and Clemmons DR (1991) Evaluation of the developmental and nutritional changes in porcine IGFBP-1 and 2 in serum by immunoassay. *Endocrinology* **129**: 2631–2638

Michell NP, Eggo MC and Langman MJS (1995) The role of IGFs and their binding proteins in colonic cancer cell proliferation. A507 *American Gastroenterological Society meeting*, 15 May 1995. *Gastroenterology* **108**: A507

Moyer MP (1983) Culture of human gastrointestinal epithelial cells. *Proc Soc Exp Biol Med* **174**: 12–15

Nam TJ, Busby WH and Clemmons DR (1994) Human fibroblasts secrete a serine protease that cleaves insulin-like growth factor binding protein 5. *Endocrinology* **135**: 1385–1391

Rechler MM (1994) Insulin-like growth factor binding proteins. *Vit Hormones* **47**: 1–114

Reeve AE, Eccles MR, Wikins RJ, Bell GI and Millow LJ (1985) Expression of IGF-II transcripts in Wilm's tumour. *Nature* **317**: 258–261

Singh A, Hamilton-Fairley D and Koistenen R (1990) The effects of IGF type I and insulin on the secretion of sex hormone binding globulin and IGF-I binding proteins by human hepatoma cells. *J Endocrinol* **14**: R1

Singh P, Dai B, Yallampalli C and Xu Z (1995) Expression of IGF-II and IGF-binding proteins in relation to growth response to IGFs. *Am J Physiol* **267**: G608–617

Tricoli JV, Rall LB, Karakousis CP, Herera L, Petrelli NJ, Bell GI and Shows TB (1986) Enhanced levels of IGF mRNA in human colon carcinomas and liposarcomas. *Cancer Res* **46**: 6119–6123

Truleau C, De Grouchy J and Chavin-Colin F (1984) Trisomy 11 p15 and Beckwith-Weideman syndrome: a report of 2 cases. *Human Genetics* **67**: 219–221

Weidemann HR (1983) Tumours and hemihypertrophy associated with Weidemann-Beckwith syndrome. *Eur J Paediatr* **141**: 129–136

© Cancer Research Campaign 1997